# Detection of domestic violence by community mental health teams: a multi-center, cluster randomized controlled trial

**DOI:** 10.1186/s12888-017-1399-7

**Published:** 2017-08-07

**Authors:** Roos E. Ruijne, Louise M. Howard, Kylee Trevillion, Femke E. Jongejan, Carlo Garofalo, Stefan Bogaerts, Cornelis L. Mulder, Astrid M. Kamperman

**Affiliations:** 1 Department of Psychiatry Erasmus Medical Center, Espri Epidemiological and Social Research Institute, ‘s Gravendijkwal 230, 3015 CE Rotterdam, The Netherlands; 2 King’s College London, Section of Women’s Mental Health, PO31 King’s College London, De Crespigny Park, London, SE5 8AF UK; 3 Department of Developmental Psychology, Tilburg school of Social and Behavioral Sciences, Tilburg University, Professor Cobbenhagenlaan, Simon Building, 5037 AB Tilburg, The Netherlands; 4 Fivoor/FPC Kijvelanden, Kijvelandsekade 1, 3172 AB Poortugaal, the Netherlands

**Keywords:** Psychiatry, Domestic violence, Intervention, Victimization, Detection, Patients, Outpatient, Community mental health teams, Randomized clinical trial

## Abstract

**Background:**

Domestic Violence and Abuse (DVA) is associated with a range of psychosocial and mental health problems. Having a psychiatric illness increases likelihood of being a victim of DVA. Despite the evidence of a high risk for DVA and the serious effects of violent victimization in psychiatric patients, detection rates are low and responses are inadequate. The aim of the BRAVE (Better Reduction trough Assessment of Violence and Evaluation) study is to improve detection of and response to DVA in psychiatric patients. In this article, we present the protocol of the BRAVE study which follows the SPIRIT guidelines.

**Methods:**

The BRAVE study is a cluster randomized controlled trial. We will include 24 community mental health teams from Rotterdam and The Hague. Twelve teams will provide care as usual and 12 teams will receive the intervention. The intervention consists of 1) a knowledge and skills training for mental health professionals about DVA, 2) a knowledge and skills training of DVA professionals about mental illness, 3) provision and implementation of a referral pathway between community mental health and DVA services. The follow up period is 12 months. Our primary outcome is the rate of detected cases of recent or any history of DVA in patients per team in 12 months**.** Detection rates are obtained through a systematic search in electronic patient files. Our secondary aims are to obtain information about the gain and sustainability of knowledge on DVA in mental health professionals, and to obtain insight into the feasibility, sustainability and acceptability of the intervention. Data on our secondary aims will be obtained through structured in depth interviews and a questionnaire on knowledge and attitudes on DVA.

**Discussion:**

This study is the first cluster randomized controlled trial to target both male and female psychiatric patients that experience DVA, using an intervention that involves training of professionals. We expect the rate of detected cases of DVA to increase in the intervention teams. With early detection of victimization of DVA in psychiatric patients we hope to improve the mental health of psychiatric patients in the short and long term.

**Trial registration:**

ISRCTN:14115257. Date of registration: 15th January 2015.

## Background

Domestic Violence and Abuse (DVA) is a major public health concern which affects the lives of people across the globe [1]. In the Netherlands approximately 45% of all persons experience at least one form of DVA during their life time and estimates suggest that approximately 200.000 Dutch persons per year are victimized by an intimate partner or family member [2]. DVA is the most prevalent form of violence and affects men, women and children in all segments of society.

The definition of DVA can vary. DVA can be defined as ‘any incident of threatening behaviour, violence or abuse (psychological, physical, sexual, financial or emotional) between adults who are or have been an intimate partner, family member, friend or otherwise closely related (i.e. a care taker or roommate) [3].

In addition to immediate physical injuries and long term conditions such as chronic pain (e.g. back or neck), gynaecologic problems such as chronic pelvic pain or sexually-transmitted diseases and hypertension [4–11], DVA is associated with a range of psychosocial and mental health problems, including loneliness, anxiety, depression, post-traumatic stress disorders, substance abuse and psychosis which can be transient as well as chronic [4, 12–15] [16–18].

Research has found a high prevalence of DVA among psychiatric patients [19] and, across all diagnostic categories, an increased likelihood of being a victim of DVA among women and men with psychiatric disorders [13, 20]. Results from the Dutch nationwide study on victimization in psychiatric patients supports the results and mentioned reports a 6-fold increase of DVA in severely mentally ill (SMI) patients as compared to the general population [20, 21].

Although in the majority of cases of DVA men are perpetrators and women are victims, in the last decade an increasing amount of research showed that, at least among psychiatric patients, men are often victims of DVA as well [22–24]. In other words, despite the absolute DVA prevalence in males is lower compared to females, in psychiatric patients this gender difference is greatly reduced. In patients the risk among men increases steeply compared to the general population, more so than in female psychiatric patients [13, 19, 21, 25]. This phenomenon is mostly overlooked.

In addition because of the social stigma associated with (male) victims of DV and the few studies on male victimization of DVA, under detection in general but in males specifically, is likely as well.

Despite the evidence of a high risk for DVA in psychiatric patients and its serious consequences on patients and their families, detection rates of DVA remain low. Around 10–30% of cases are detected by psychiatric service providers [26]. Clinicians often report barriers to ask for experiences of concurrent DVA. These barriers include the fear to confront and offend patients or the fear to induce false memories [27], and perceived lack of knowledge and expertise to address the abuse [28]. Moreover, after the detection of DVA, treatment plans rarely take these experiences of DVA into account [25]. These figures are alarming and indicate that a great need to invest in interventions to improve the identification of DVA victimization and provision of mental health services addressing the prevention of DVA (re-)victimization among psychiatric patients. Although there is little research on this subject, some have suggested the use of an individual screenings tool to improve detection of and outcomes for domestic violence in women [29]. However, a Cochrane systematic review of screening in primary care found that this did not increase referrals to services specialized in DVA nor did it decrease experienced DVA in victims [29]. These results suggest that information dissemination on DVA to professionals and patients and a screenings tool for DVA in isolation does not create consistent, sustainable improvements in identification and response to DVA.

As was shown in studies [13, 30], identification rate of victims of DVA by health care professionals can increase with a sustainable training of professionals and changes in policy including the implementation of a referral and care pathway within health care systems. Apart from a positive effect on detection rate, these type of interventions result in more referrals to specialist services for DVA [13, 30].

The BRAVE study is informed by the Linking Abuse and Recovery through Advocacy (LARA) pilot study in mental health services and Identification and Referral to Improve Safety (IRIS) cluster trial in primary care conducted in the United Kingdom [13, 30], as both of these studies showed promising results. Detection and referral rates increased after a multi-faceted intervention was provided to clinical teams which included training by the DVA sector and a referral and care pathway which included referral to the DVA sector; the level of functioning and abuse experienced by psychiatric patients in the LARA study was measured and also improved [31]. The BRAVE study is the first randomized controlled trial which focuses on males and females who have a mental illness.

The BRAVE study is an interventional study. The intervention consists of: 1) a knowledge and skills training for mental health professionals about DVA, 2) knowledge and skills training of DVA practitioners about mental illness, 3) the provision and implementation of a direct referral pathway between community mental health services and DVA services for victims of DVA with mental illnesses. The effect of this intervention will be tested in a cluster randomized controlled trial comparing the detection rate of domestic violence in psychiatric patients of community mental health teams in the intervention condition versus teams in the control condition. Furthermore we aim to assess the feasibility, sustainability and acceptance of the intervention for clinicians, policy makers, managers and victims of DVA in both the community mental health services and the DVA services. We expect that the number of detected cases of DVA will increase in the intervention group compared to the control group.

## Methods

### Domestic violence and abuse services in context of the public health system in the Netherlands

In the Netherlands, domestic violence and abuse (DVA) services are part of the public health system. The public health system is financed by government, and services are free of charge for every citizen. The public health services cover a wide range of problems, such as domestic violence, youth work, and housing. Public health services are the responsibility of the municipality. With regards to DVA, starting from 2015, every municipality in the Netherlands has installed a helpline service, for both professionals and victims of DVA, named Veilig Thuis (Safe Home).

A Veilig Thuis-service functions as a gatekeeper. They assess cases of (suspected) DVA, provide advice in cases of (suspected) DVA and coordinate referrals to third party services on DVA. Veilig Thuis (VT) covers both child abuse and intimate partner violence.

All municipal VT-services adhere to national DVA guidelines (diagnostic and referral guidelines). These guidelines are general in nature, which means that slight differences in interpretation and execution may exist between municipalities.

Thus, the municipal care for victims of DVA offers the following services [32].

Informal advice:

- Assessment: if a person calls the helpline, VT will assess the situation during that phone conversation. The helpline service is available for everyone with a concern regarding DVA. This could be, for example, a co-worker, victim of DVA or a clinician.
- Advice: after assessment VT will offer advice to whomever using the helpline. This advice ranges from passively monitoring the situation to notifying the police because of a (potentially) dangerous situation. VT can also give referral advice.

Formal advice:

- Assessment: the helpline can also be used for (health care) professionals who want to ask for a formal advice in a case of (suspected) DVA from VT. This means that VT will be officially involved, they will assess the situation in person and provide an official report after their assessment which includes directives for specialized services.
- Referral: after assessment, VT will refer the case for the care they deem necessary to stop the violence. This care can vary from empowerment courses delivered by third party DVA services or social work services to temporary eviction of the perpetrator. If a case is deemed dangerous, VT can directly report the case to the police and safeguard the victim through admission in a safe house.

VT employs a variety of professionals namely, doctors specialized in DVA behavioral experts, nurses. Most employees answering the helpline are social workers.

#### Legislation

All professionals working in health care are encouraged to, and in the case of child abuse are obliged to, follow a protocol on (suspicion of) DVA by law. This protocol is called ‘Meldcode’ [33]. Our aim is to improve and further implement this protocol in daily practice as a part of the referral pathway. The Meldcode consists of 5 steps a health care professional should follow when DVA is suspected. The steps are: 1) screening for possible signals of DVA, 2) consultation of a colleague or a DVA professional working at VT, 3) discuss suspicion of DVA with the client/patient, 4) assessment of the severity of the violence. In this step a consultation of a DVA professional at VT is possible, 5) patient referral to a service specializing in DVA (if deemed appropriate) or a formal assessment from VT. If at step 5 the health care professional decides to formally ask advice from VT, VT will assess the situation and refer to/involve the care that they think is needed to stop the violence.

### Study setting

The study will be conducted in the municipalities of Rotterdam and The Hague, the Netherlands. DVA services provided in these municipalities are provided by VT Rotterdam and VT The Hague (see previous paragraph for details). Mental health care in these municipalities is provided by two big community mental health clinics (CMH) i.e. BAVO Europoort (Rotterdam) and Parnassia The Hague. These clinics provide both out-patient and in-patient mental health care and cover the Rotterdam- Rijnmond and the Hague area, which inhabits approximately 2.5 million people. Service use is free of charge for all citizens with health insurance, which is obligatory in the Netherlands, after paying a limited contribution with a maximum of 385 euros per year.

### Ethics

This design takes into account some important dilemmas with regards to studying a population at risk for DVA. In this study, screening for domestic violence is an integral part of the intervention, because it is considered unethical to screen for domestic violence while not offering any kind of help to positively screened patients [34].

There is a high level of acceptance for screening among women with severe mental illness [13, 28]. However, we need to protect professionals and patients from violence as a consequence of disclosure of domestic violence by offering skills and information on how to assess risk of and respond to violence. This theme will be a substantial part of the training of professional and intervention of the patient. Adverse events will be reported to the researcher and scientific committee.

Professionals interested in taking part in the in-depth interviews will be asked to sign an informed consent prior to the interview. Professionals will be recruited by the researcher.

Patients will be asked through their clinicians to participate in an in-depth interview. If patients are interested they will receive an information letter about the study and a researcher will approach the patient to obtain informed consent.

With regards to our primary parameters, data will be collected on team level, and cannot be traced back to individual patients nor clinicians. Data of patients within teams will be stored and analysed anonymously. With regards to our secondary parameters, data on individual professionals (the data of the short questionnaire on DVA and the transcripts of the semi-structured interviews) will be linked to subjects using an identification number. The code list will be stored separately from the anonymous research data, and secured by a password. All data will be stored on local servers in designated folders. Only the research group will have access to these folders. Data will be handled by the research group only.

Due to the nature of this study, there will be no specific post-trial care. We do strive however, to implement champions of DVA in each intervention team and support meetings where participants can ask questions about complicated cases of DVA. While we strive to set up support meetings, these meetings fall beyond the scope of this study.

The Medical Ethical Committee of the Erasmus MC Rotterdam approved the BRAVE study. All amendments will also be approved by the Medical Ethical Committee of the Erasmus MC Rotterdam before publication of any published articles.

### Dissemination

Trial results will be published in scientific journals. Additionally main results will be communicated to the participating mental health institutions. The trial is listed on the ISRCTN registry with study ID ISRCTN14115257.

### Study design

This study is a cluster randomized controlled trial. Clusters in this trial are CMH teams working according to the Flexible Assertive Community Treatment methodology (FACT) [35].CMH teams provide outreaching care to patients with severe mental illness (SMI). A CMH team consists of approximately 10 mental health professionals including a psychiatrist, psychologist, social psychiatric nurses and social workers. Their caseloads consists mostly of patients with schizophrenia, psychosis, bipolar disorder or chronic depression. The level of intensity of care can vary over time and from patient to patient. Each CMH team provides outreaching care for patients living in a certain district of The Hague or the Rotterdam – Rijnmond area. The districts in this study are defined through postal codes. CMH teams included in this study provide care for approximately 200–250 patients.

### Eligibility criteria for CMH teams

Subjects (teams) which meet any of the following criteria will be excluded from participation in this study:

- Teams providing care to patients <18 years
- Teams with more than 20% of the employees working over different teams
- Teams specialized in one specific mental illness (e.g. autism)
- Teams without a functioning electronic patient file system or with <12 months of historical data at the start of the intervention.

### Sample size

With regards to our primary research aim, we will test the hypothesis that the intervention condition will result in a significantly higher rate of detected cases of DVA compared to the control condition over a 12 month follow up period.

To calculate the sample size needed in this cluster RCT, we started with a sample size calculation assuming individual randomization and inflated this number by a design effect to account for randomization by cluster [36]. This inflation factor (IF) is dependent on average cluster size, and the intra-class correlation (ICC). Using this calculation we found that with a minimum of 12 intervention teams and 12 control teams, with the assumption of a detection rate of 0.25% in control teams (based on the LARA study [13]) and an ICC of 0.03 (based the IRIS study [30]) we will be able to detect a three- to four-fold increase (Beta = 3.55) in the detection rate with a power of 80% and a significance level of 0.05. This calculation assumes an average of 200 patients in every team.

Regarding knowledge and attitudes on DVA, a sample size of 24 teams allows us to detect a large sized increase of knowledge between the intervention and control teams.

In order to answer Aim 2 we will ask a purposeful sample of persons from every profession involved in the study for an in depth interview about the feasibility, sustainability and acceptance of the intervention. A minimum of ten interviewees will be selected from the DVA services, and ten from CMH services. Furthermore all trainers in this study will be interviewed on their experiences. Additionally, we will interview a total of 30 patients or more until saturation of the themes is achieved.

### Recruitment

The CMH teams eligible for inclusion will be appointed by the management of the participating facilities to participate in the trail.

Recruitment regarding the in-depth interviews on feasibility, sustainability and acceptability will be done by the research team. All professionals participating in the study will be asked if they want to participate in an interview. Patients will be approached through their primary clinician at the CMH team. If a patient is interested they can contact the research team for further information and possible participation. Additionally we will recruit patients through patient support organizations, support groups for victims of DVA, and advertisements placed at the locations of the CMH services. Interested patients will be provided with an information letter on the study. After signing informed consent the patient will approached for an interview. We will interview patients until saturation of the themes is achieved. Patients will receive a fee of 20 euros after the interview.

### Randomization and blinding

We will randomly allocate the 24 CMH teams in a 1:1 allocation ratio to receive either no intervention or the BRAVE intervention. Randomization will be conducted using a web-based computer-generated randomization schedule ten-ALEA (randomization software programme, version 2.2), using block sizes of 2. Social-economic status of the service area of the CMH team dichotomized into high and low social-economic status, will be used as stratification factor. Allocation will be facilitated by an independent researcher. Due to the nature of this intervention, it is not possible to blind the CMH professionals of the teams for allocation status. An independent researcher, blinded for allocation, will be responsible for extracting the primary data fields regarding the primary outcome measure (e.g. the number of screened DVA cases per team) from the electronic patient files.

### Intervention

The intervention consists of 1) knowledge and skills training for mental health professionals of the CMH teams about DVA, 2) knowledge and skills training of DVA practitioners about mental illness, 3) the provision and implementation of a direct care pathway between CMH services and DVA services for victims of DVA with mental illness.

1. *Knowledge and skills training for mental health professionals about DVA:*

The training for the teams has been developed by the research team and is aimed at improving knowledge and skills regarding detection and management of DVA. At the start of the trial, all CMH teams in the intervention group will receive a training of approximately 8 hour in total, divided over 2 days. The topics covered in the training used in the LARA-intervention were included here [13] and consist of four main themes namely;

- Improving knowledge about DVA; (the definition and different forms of DVA; prevalence and incidence of DVA in the Netherlands; and symptoms and signals of DVA, including cultural differences in expression).
- Identifying and documentation of DVA when taking the patient’s history: this theme will cover different techniques a clinician can use to discuss and assess DVA with a patient and includes advice on how to document DVA.
- Safety: this theme entails safety assessment and management for patients and clinicians and will also include laws and legislation on DVA in the Netherlands.
- Treatment/follow up: this theme will discuss referral pathways and ways to empower/support patients who are victims of DVA.
  The training is modified according to the characteristics of the specific municipality. This means that the teams will receive training with a referral pathway adapted to the Rotterdam and The Hague municipality, respectively.

### Implementation

All members of a CMH team randomized in the intervention condition will be invited to attend the training. Attendance will be monitored. During, and after initial training, teams are actively encouraged to implement the techniques learned in their daily practice.

After the training, clinicians will receive an extended manual incorporating good practice guidelines and information on local/national DVA services. To improve implementation of the knowledge gained in the training, each team in the intervention condition will be recommended to assign a advocate on DVA. Advocates in the intervention teams will collect all problems and questions regarding DVA in their team. We will maintain close contact with the DVA advocates throughout the study through e-mail. Advocates can address problems in the collaboration with DVA practitioners and/or referral to DVA services to the DVA expert team in the institution. This expert team is not part of this study and is already in place at the two participating institutions before the beginning of this study. We will also hand out posters with conversational techniques on DVA and cards team members can use as an aid when talking to their patients about DVA.

The teams in the control condition will not receive any additional training in DVA. Clinicians in the control condition however, can refer patients to DVA services(according to standard practice), when they detect a patient suffering from DVA.

2) *Knowledge and skills training of DVA practitioners about mental illness:*

The professionals working at VT will be offered training on mental illness and procedures within mental health care. The training has been developed by the research team and can be adjusted according to the baseline level of knowledge of the professionals working at VT. Regardless of adjustment, the training consists of a the following key elements:

- Knowledge on mental illness (including conversational techniques and pitfalls and possibilities in dealing with psychiatric illness)
- Structure of mental health care services and patient-doctor confidentiality
- Referral to and communication with clinicians working in mental health care

3) *The provision and implementation of a direct care pathway between CMH services and DVA services for victims of DVA with mental illness*

Our study will focus on providing professionals working in mental health care with the necessary skills refer victims of DVA to services specializing in DVA and to implement the Meldcode protocol as part of the referral pathway in daily practice. The Meldcode is explained in more detail in the section legislation. Professionals will be provided with a visual outprint of the Meldcode, contact information on local DVA services and information on resources on DVA in their own organization in the manual of the training. Every professional has access to a work related smartphone and professionals will be encouraged to install the Meldcode app.

### Outcome measures, aims and data collection

#### Primary outcome

The primary outcome measure is the rate of detected of DVA cases per CMH-team, taking into account the number of patients in each team. Outcomes will be obtained through a systematic search in electronic patient files at the participating mental health institutions. We will search the electronic patient files using a set of keywords, their synonyms, (miss-)spellings and words closely associated with DVA. Our main keyword will be domestic violence. This keyword will first be piloted and validated. A specialized software program will be used to analyze the information in the electronic patient files of the detected cases to determine words closely associated with domestic violence. To validate the detected cases, we will assess each detected case of DVA individually using information from the electronic patient file. Non-cases will be verified randomly. Reliability of the data extraction will be evaluated by repeating the data extraction on a subset of teams by a second independent researcher and compare the outcomes. To ensure comparability in timing, we will pair control and intervention teams and obtain outcome data simultaneously. We expect an underreporting of detected cases and over-reporting of referred cases by the clinicians in the patient records [30]. Since these biases will affect both intervention and control condition equally, study results will remain valid.

#### Secondary aims and outcomes

The BRAVE intervention has the following secondary aims and outcomes:

Aim 1 - to assess gains and sustainability in knowledge on DVA among mental health professionals.

Aim 2 - to assess the feasibility, sustainability and acceptance of the intervention among CMH teams and,

Aim 3 - to assess the (possible) referral pathway to DVA services and the implementation of the Meldcode.

We will assess Aim 1 using a structured questionnaire – The BRAVE Survey - developed specifically by the study team, to assess CMH teams knowledge and attitudes regarding DVA.

Aim 1 - the BRAVE survey

The BRAVE survey was informed by the PREMIS [37] and PROTECT questionnaires [38].

All members of the included teams will receive this survey. The survey has a total of 53 questions divided into 5 sections: (1) Respondent profile, (2) Previous Courses on DVA (3) Skills in management of DVA, (4) Knowledge on DVA and (5) Opinions on DVA. Section 1; “Respondent profile” consists of 7 questions, of which 3 are multiple choice and 4 questions an individual can fill in. Participants are asked about their professional background (work experience, current profession, educational level) and general information (date, age, gender); section 2; “Previous courses attended on DVA” consists of a multiple choice question about previous courses on DVA. If participants check yes they are asked 4 questions about duration of this course and when this course was completed. Section 3; “Skills in management of DVA” consists out of 7 multiple choice statements. Participants can check the box on the Likert scales, ranging from 1 (not skilled) to 5 (very skilled), the extent to which they feel skilled to handle/judge a particular described situation regarding DVA. Section 4; “Knowledge on DVA” consists of a total of 14 multiple choice statements. For the first three questions, participants need to check the box on Likert scales, ranging from 1 (nothing) to 5 (a lot). These questions are followed by 10 multiple choice questions with statements about DVA where participants can choose between true (1) and not true (2). The last question in this section asks the participants to combine the correct described statement with the right stage of the process of discontinuing a relationship with a perpetrator. Section 5; “Opinions on DVA” consists of 16 statements on DVA where participants can choose whether they agree on a Likert scale ranging from 1 (do not agree) to 5 (agree).

To be able to compare the scores of the survey, sections 3 (Skills), 4 (Knowledge) and 5 (Opinions) will be calculated separately per participant and will then be aggregated at a team level. Qualitative answers will be given a score and, post-hoc assigned to answering categories. Statistical comparisons will be made between intervention and control teams.

This questionnaire will be assessed at baseline, after 6 months of the intervention and after 12 months of the intervention.

We will assess Aim 2 by conducting semi structured in-depth interviews with members of CMH teams, policy makers, patients and DVA practitioners about the feasibility, sustainability and acceptance of the intervention.

Aim 2 Semi-structured in depth interviews

We will obtain outcomes regarding opinions on feasibility, sustainability and acceptance of the intervention using a semi-structured in depth interview with a purposive sample of professionals in the intervention - and control group, team-leaders, managers, patients in the intervention group, patients in the control group, patients who have experienced DVA and patients who didn’t experience DVA. The interview for clinicians will mostly focus on their opinions and personal definition of the following themes:

- DVA
- actions (not) taken
- referral pathways between CMH services and DVA services
- impact of the intervention on care provision and care professionals (including personal safety)
- impact of the intervention on client relationship
- impact of the intervention on client wellbeing (including personal safety)
- level of implementation of the intervention in daily practice
- feasibility of implementation of the intervention
- sustainability of the intervention

In the policy makers of the included mental health institution and team managers the in depth interview will focus on their opinions and personal definition of the following themes:

- DVA
- the intervention
- referral pathways between CMH and DVA services
- level of collaboration between CMH and DVA services
- impact of the intervention on care provision and operational capability
- cost-effectiveness of the intervention

The in depth interview with psychiatric patients will focus on their opinions and personal definition of the following themes:

- DVA
- disclosure of DVA
- available help for DVA

We will gather data until saturation of the theoretical framework (what is the feasibility, sustainability and acceptance of the intervention as well as how was the intervention implemented). Interviews will be recorded and transcribed as preparation for analysis. We will analyze the data with a mix of content and framework analysis. After the frameworks are identified, the data will be structured, labelled and coded.

We will assess aim 3 using the detected cases from the primary outcome. We will then search electronic patient files of the detected cases to identify cases of DVA referred to professional DVA services per included CMH team.

Aim 3 Evaluation of the referral pathway to professional services on DVA of detected cases

Detected cases are extracted from the electronic patient’s files using the method described in the section ‘Primary outcome’ of this article. Of all detected cases of DVA the following information, will be extracted from the electronic patient file, when available: 1) the time from disclosure of DVA to referral, 2) details on any consultation with VT and/or colleagues, including the possible reason for consultation and advice given; 3) if the patient was involved in the decision to engage with VT and/or colleagues and their opinion about the given advice; 4) how the severity of the DVA case was assessed and which sources were used for the assessments; 5) details on the referral process, Including the reasons for the formal request for advice/ informal advice from VT, reasons for the ultimate decision to refer/not to refer, details of the referral site, the care provided at the referred institution, and outcomes for the patient with regards to the (ending of) violence. In doing so, we aim to assess the qualitative characteristics of the referral pathway in the intervention and control teams.

### Statistical methods

#### The number of DVA cases detected per team

We will use Generalized Estimating Equations analysis to compare detection rate in the intervention and control teams. Independent variables will be number of detections each CMH team over 12 month follow-up period. The number of patients in each CMH team will be included as denominator, and CMH team will be included as a random effect to take the clustered nature of the data into account. Subsequent analysis will be adjusted for the number of detected cases at baseline. Sensitivity analyses will be conducted to evaluate the impact of team characteristics (such as case load size, proportion of clinicians trained on DVA, and proportion of female patients) on the intervention outcomes. Due to the nature of our study, we will not have any missing data.

#### Survey on knowledge and attitudes on DVA

Subscale scores from BRAVE survey will be calculated. For each participant change scores will be calculated between baseline and follow-up score. Generalized Estimating Equations analysis will be used to compare mean change score in the intervention and control teams. The CMH team will be included as a random effect to account for the clustering of the data. Questionnaires with more than 5% of the questions missing will be removed from the sample. Questionnaires with less than 5% missing will be imputed using multiple imputation.

### Fidelity scale

Fidelity of the BRAVE intervention will be assessed by an independent researcher during the intervention at the time points of 6 and 12 months. A fidelity scale will assess whether 5 key components of the intervention are in place 1) training for mental health professionals on DVA, 2) training for DVA practitioners on mental health, 3) identification of the components of DVA in routine mental health care, 4) Integrated DVA referral and care pathway 5) collaboration between DVA specialists and mental health professionals. The fidelity scale was developed in collaboration with the LARA research group.

### Data monitoring

This is a low risk study, a full data monitoring committee is not deemed necessary. A monitor and data archiving plan is in place.

### Assessment schedule

All teams eligible for participation will be included before the start of the intervention. After inclusion, teams will be assigned randomly to the control condition and intervention condition. All teams will receive a baseline assessment, an assessment at 6 months and an assessment at 12 months using the BRAVE survey. The number of detected cases of DVA will be studied over a 1 year period. The in-depth interviews will be conducted in the last period of the intervention period and after the intervention period (Table 1).

**Table 1:**
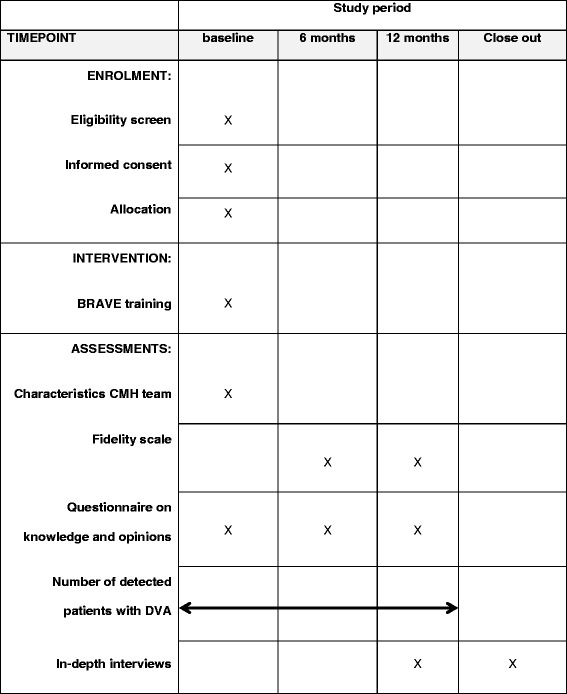
An overview of activities during the study period

## Discussion

DVA is a problem worldwide and particularly vulnerable groups, such as psychiatric patients with severe mental illness, become victims of DVA while at the same time DVA often remains undetected in this group by mental health professionals. In this protocol, we will measure the efficacy of an intervention to improve knowledge and skill training for professionals about DVA and mental illness, as well as to implement a referral pathway between CMH and DVA services for DVA victims with mental illness. According to these premises, we underline the importance of this intervention for psychiatric patients suffering from DVA.

This study is the first cluster randomized controlled trial targeting both male and female psychiatric patients who potentially experience DVA, using an intervention that involves a training of mental health and DVA services, and the focus on the implementation of a referral protocol. Among victims of DVA, loss to follow up is considered a risk. MacMillan et al. [34] reported 42% loss over an 18 months follow up period. This high percentage loss is partly due to the nature of the problem studied. Victims of DVA show a high level of mobility and might be at high risk for subsequent violence after disclosure of the DV. By assessing our primary outcomes at team level, we minimize biases caused by individual drop out.

Furthermore, by randomizing teams we avoid contamination of skills and techniques of professionals in the intervention condition to the professionals in the control condition. However, since a cluster RCT is less precise than an individual trail, the chance of failing to detect a true intervention effect might increase (type II error). Also, the number of clusters in this trail is relatively small (i.e. 24 clusters), therefore randomization might partly fail, which would cause the patients in the control and intervention condition to differ from each other [39].

We do not foresee many risks in the attainment of this study. However, possible risks could occur if the mental health institutions discontinue facilitation of the CMH teams or reorganize the team structure, for instance by merging existing teams during the study period. This will affect the number, size and composition of clusters. This might lead to contamination and decrease of power. Also, municipal reorganization of the DVA services might affect the attainment of this study.

With better detection rates of DVA, the focus of research on this topic could shift towards improvement of treatment, prevention and interventions on DVA on an individual level.

## Abbreviations

BRAVE: Better reduction through assessment of violence and evaluation
CMH: Community mental health
DVA: Domestic Violence and Abuse
FACT: Functional assertive community treatment
ICC: Intraclass Correlation
IF: Inflation factor
IRIS: Identification and Referral to Improve Safety
LARA: Linking Abuse and Recovery through Advocacy
MEC: Medical Ethical Committee
RCT: Randomized controlled trial
SMI: Severe mental illness
VT: Veilig Thuis
